# Total flavones of *abelmoschus manihot* enhances angiogenic ability both *in vitro* and *in vivo*

**DOI:** 10.18632/oncotarget.19264

**Published:** 2017-07-15

**Authors:** Lingyi Tang, Wu Pan, Guisong Zhu, Zhihui Liu, Dongling Lv, Meng Jiang

**Affiliations:** ^1^ Affiliated Hospital of Nanjing University of Chinese Medicine, Nanjing, Jiangsu 210029, PR China; ^2^ Nanjing University of Chinese Medicine, Nanjing, Jiangsu 210029, PR China

**Keywords:** abelmoschus, chorioallantoic membrane, flavones, human umbilical vein endothelial cells, neovascularization

## Abstract

Angiogenesis is a process of new blood vessel formation from pre-existing vessels. It is a normal and vital process in growth and development, as well as in wound healing and in the formation of granulation tissue. Total flavones of *Abelmoschus manihot* (TFA) are the major constituents of the traditional Chinese herb *Abelmoschus manihot* L. Medic. The aim of this study is to investigate the effect of TFA on angiogenic ability using human umbilical vein endothelial cells (HUVECs) *in vitro* and chick chorioallantoic membrane (CAM) *in vivo*. HUVECs were treated with TFA at different concentrations. Cell viability, cell cycle progression, cell apoptosis, cell migration and tubular formation were investigated. The expression of vascular endothelial growth factor (VEGF) and kinase insert domain receptor (KDR, VEGFR-2) was examined by immunohistochemistry to identify mechanism of action of TFA. CAM model was used to evaluate the effect of TFA on angiogenesis *in vivo*. Our results showed that TFA promoted HUVECs proliferation in a dose- and time-dependent manner. It increased HUVECs migratory ability and the number of tubular structure, promoted vessel formation in HUVECs culture and CAM model. Furthermore, TFA treatment resulted in a decrease in cell apoptosis and enhanced the expression of VEGF and KDR. Taken together, TFA, as the major active component isolated from the traditional Chinese herb *Abelmoschus manihot* L. Medic, could enhance angiogenic ability of HUVECs *in vitro* and CAM *in vivo*. TFA may be used in the treatment of wound healing and ischemic/reperfusion injuries.

## INTRODUCTION

Angiogenesis is a complex process involving basement membrane degradation, endothelial cell proliferation, migration and tube formation [1]. It is a normal and vital process in growth and development, as well as in wound healing and in the formation of granulation tissues [2, 3]. Angiogenesis is also involved in the etiology of many diseases, such as psoriasis, diabetic retinopathy, cancer, myocardial and limb ischemia [4, 5].

*Abelmoschus manihot* L. Medic, widely distributed in China and India, is a flowering plant of the mallow family Malvaceae. As a commonly used traditional Chinese herbal medicine, A. *manihot* was once recognized as “an effective herb in the treatment of various malignant sore, cellulitis and burns” [6]. In addition, according to various ancient prescriptions, A. *manihot* was used to treat carbuncle, stranguria, empyrosis, hematemesis, metrorrhagia and metrostaxis. Recent studies showed that these effects are due to the total flavones of A. *manihot* (TFA), represented by seven chemically identified flavone glycosides [7]. Pharmacological and clinical studies have lately indicated that TFA exhibit a wide spectrum of physiological activities, including protective effect on myocardial/cerebral ischemic/reperfusion injuries and inflammatory renal/hepatic injuries [8-11]. The treatment of ischemic reperfusion injuries, inflammatory injuries and wound healing is closely related with angiogenesis, which is a process of the growth of new blood vessels [12–14].

The effect of TFA on angiogenesis has not been explored. The present study was designed to investigate and validate the effect of TFA on angiogenesis and its possible mechanism of action. Human umbilical vein endothelial cells (HUVECs) and a chick chorioallantoic membrane (CAM) model were used to determine whether TFA promote angiogenesis and blood vessel regeneration.

## RESULTS

### TFA promoted HUVECs proliferation

**Figure 1:**
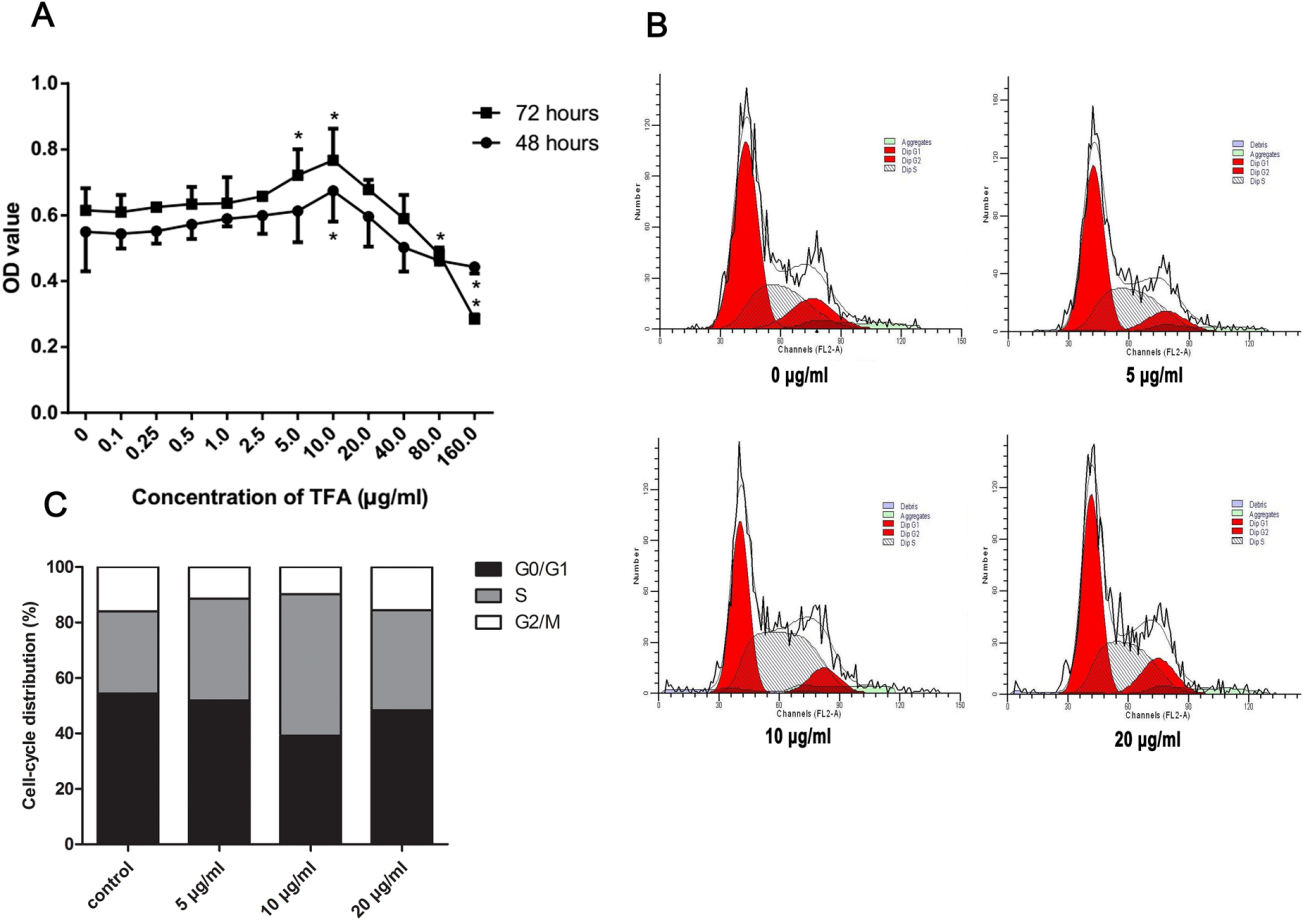
Effect of TFA on HUVECs proliferation *in vitro* **(A)** HUVECs cell viability after treatment with TFA at increasing concentrations examined by MTT at the indicated times. Data was expressed as means ± SD. *p < 0.05 compared with control group. **(B)** and **(C)** FACS images showing cell cycle of HUVECs. Cells were treated with TFA for 72 h followed by fluorescent dye staining and the percentage of cell cycle phase was shown.

Figure 1A shows the result and statistical analysis of HUVECs viability after treatment with TFA at increasing concentrations, ranging from 0.1 μg/ml to 160 μg/ml, at different time points (48 h, 72 h) by MTT assay. TFA significantly increased HUVECs cell viability at the concentrations of 5 μg/ml, 10 μg/ml compared to the control group (TFA 0 μg/ml). Moreover, as indicated from the dose-time relationship, the TFA-treated group at 10 μg/ml displayed the maximum proliferative rate at both 48 and 72 h. Based on the above results (Figure 1A), 5 μg/ml, 10 μg/ml and 20 μg/ml TFA were chosen to be the optimal concentrations for use in our studies.

The above experiment had shown that cell viability significantly increased in TFA-treated HUVECs by MTT assay. We next evaluated the cell cycle change in TFA-treated HUVECs in order to investigate TFA influence on cell cycle regulation. As shown in Figure 1B and 1C, the effect of TFA on HUVECs cell cycle was expressed by the percentage of cells in G0-G1, S, G2-M phase. A significant increase of cells in S-phase was noted after TFA treatment at 72 h. Conversely, the percentage of G0-G1, G2-M phase cells was decreased, indicating that TFA could prompt more cells into DNA synthesis phase, maintaining HUVECs in a proliferative state. Moreover, the TFA-treated group at 10 μg/ml exhibited the maximum proliferative state, which was consistent with the above MTT assay.

### TFA increased HUVECs migration

**Figure 2:**
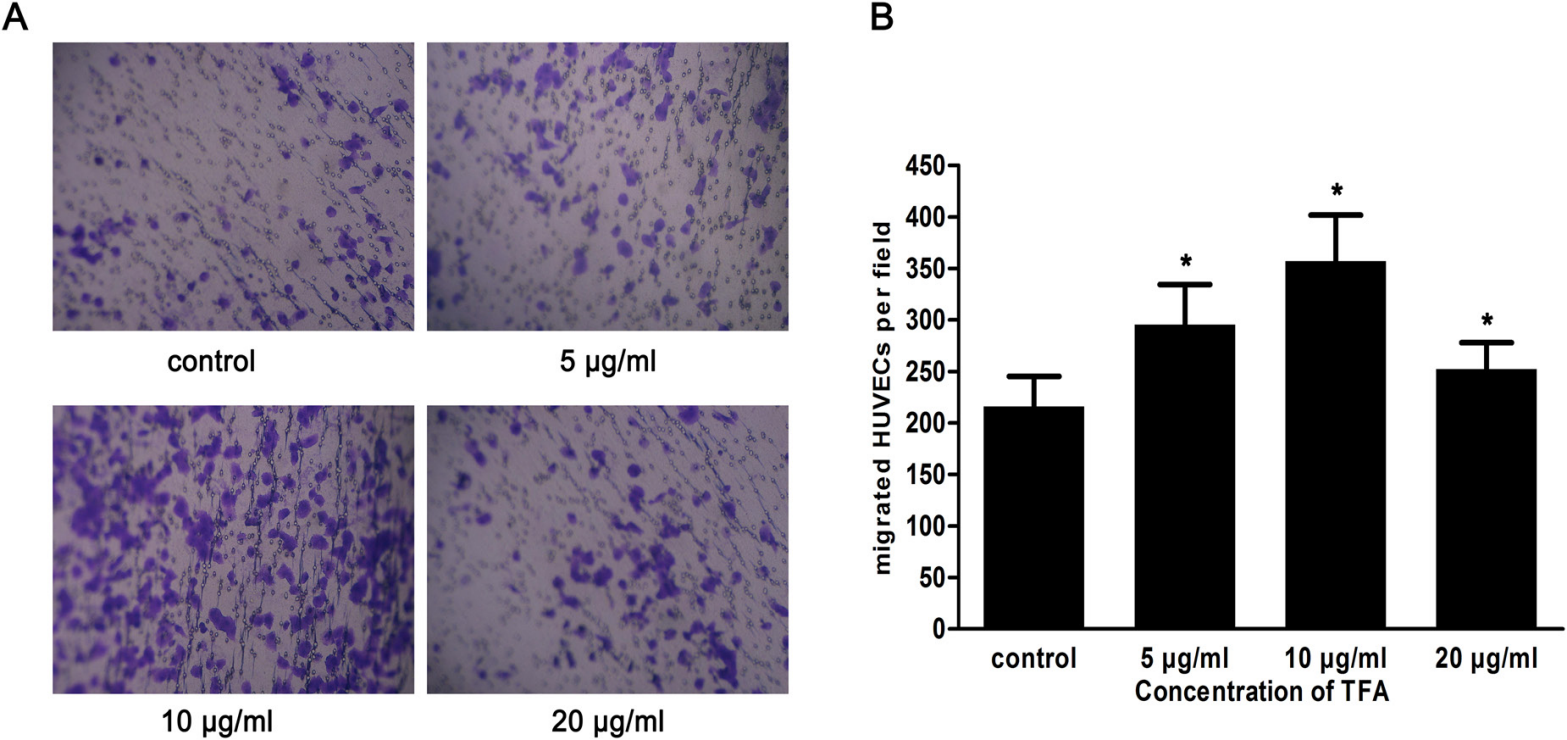
Effect of TFA at different concentrations on HUVECs migration **(A)** HUVECs were treated with TFA and the migration ability was evaluated using transwell chambers (original magnification 100×). **(B)** Quantitative results of the cells that were in the lower chamber attached to the lower side of the membrane. Data was expressed as means±SD based on three independent experiments. *p < 0.05 compared with control group.

As shown in Figure 2A and 2B, the number of cells that migrated to the lower chamber attached to the lower side of the membrane was 295.5±38.95, 357.1±44.76, 252.4±25.55 after 5 μg/ml, 10 μg/ml, 20 μg/ml TFA treatments, respectively (*p* < 0.05 vs. control), suggesting a remarkable TFA effect on HUVECs migration. Although angiogenesis is a very complex process, the migratory ability of endothelial cells is one of the most critical steps. Thus, the improved migratory ability we observed might represent a key step in TFA-promoting angiogenesis.

### TFA promoted tube formation in HUVECs

**Figure 3:**
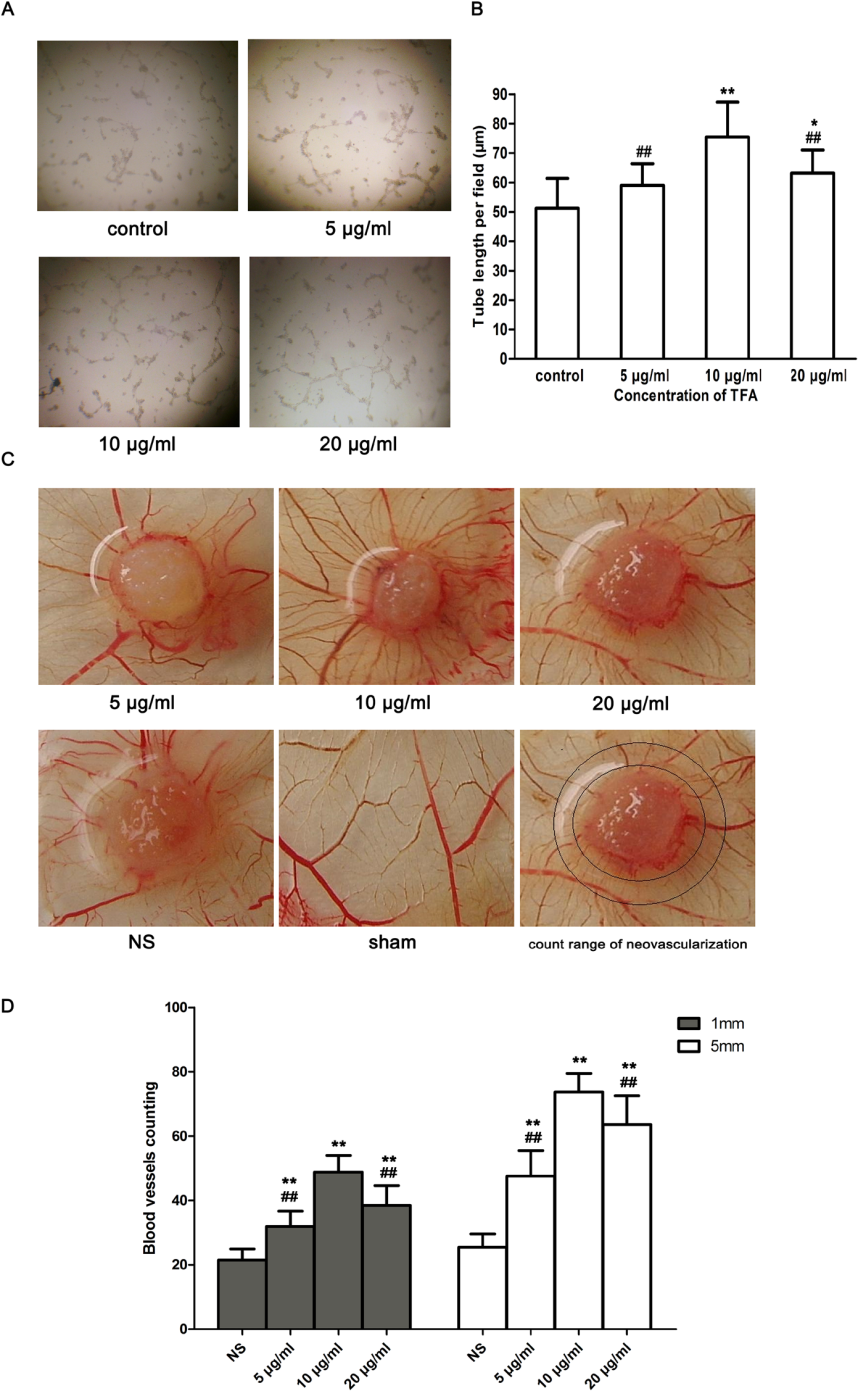
Effect of TFA on HUVECs tube formation and CAMs neovascularization **(A)** Morphological changes (tube branch and length) of HUVECs differentiation in Matrigel Matrix were observed under a phase contrast inverted microscope (100×). **(B)** Quantitative analysis of the total tube length in control and TFA-treated groups obtained by ImageJ software. Data are expressed as the means of tube length (μm)±SD. *p < 0.05, **p < 0.01 compared with control group. ##*p*<0.01 compared with TFA group at 10 μg/ml. **(C)** Effect of TFA on angiogenesis in CAM. The sham group without any treatment showed a certain rate of angiogenesis. However, neovascularization was found in CAM after incubation with TFA at different concentrations (5 μg/ml, 10 μg/ml, 20 μg/ml) for 72 h. TFA-treated groups were significantly vascularized and showed a radial growth around the carrier materials (sterile gelatin sponges), compared to control group. **(D)** Quantified neovascularization in CAM. Neovascularization was quantified around the carrier materials (sterile gelatin sponges). Marked circles represented count range of neovascularization. The inner circle was 1 mm from the edge of sterile gelatin sponge (1 mm group) while the outer circle represented 5 mm from the edge of sterile gelatin sponge (5 mm group). Data was expressed as means±SD. **p < 0.01 compared with control group. ##*p* < 0.01 compared with TFA group at 10 μg/ml.

The tube formation assay is a fast and quantifiable method for measuring *in vitro* angiogenesis [15, 16]. Tube formation occurred within hours and newly formed tubes were quantified to evaluate the ability of endothelial cells to form capillary-like structures. As shown in Figure 3A, TFA-treated groups (5 μg/ml, 10 μg/ml, 20 μg/ml) revealed the ability to form more complex and branched capillary-like structures compared with the control group in which these structures were shorter with broken tubes. Moreover, Figure 3B shows that the total tube length of control group was 51.26±10.14μm, while TFA at 5 μg/ml, 10 μg/ml and 20 μg/ml increased the length to 58.98±7.42 μm, 75.42±11.96 μm (*p* < 0.01 vs. control), 63.14±7.94 μm (*p* < 0.05 vs. control), respectively.

### TFA promoted blood vessel formation in **CAM** model

The chick chorioallantoic membrane (CAM) tends to be highly vascularized, making it a natural *in vivo* model of angiogenesis. Thus the CAM assay has been used extensively to study angiogenesis. Figure 3C shows a branching pattern of blood vessels in the sham group. However, a radial growth neovascularization around the carrier materials (sterile gelatin sponges) appeared in TFA-treated groups. The neovascularization in the TFA-treated CAMs led to the formation of more multi-stage capillaries, compared with those in the control group. In addition, the concentration of 10 μg/ml TFA led to a more abundant neovascularization and largely promoted angiogenesis both in the rings of 1 and 5 mm diameters around the gelatin sponges. Furthermore, CAM neovascularization quantification demonstrated the powerful effect of TFA on angiogenesis (Figure 3D).

### TFA decreased HUVECs apoptosis

**Figure 4:**
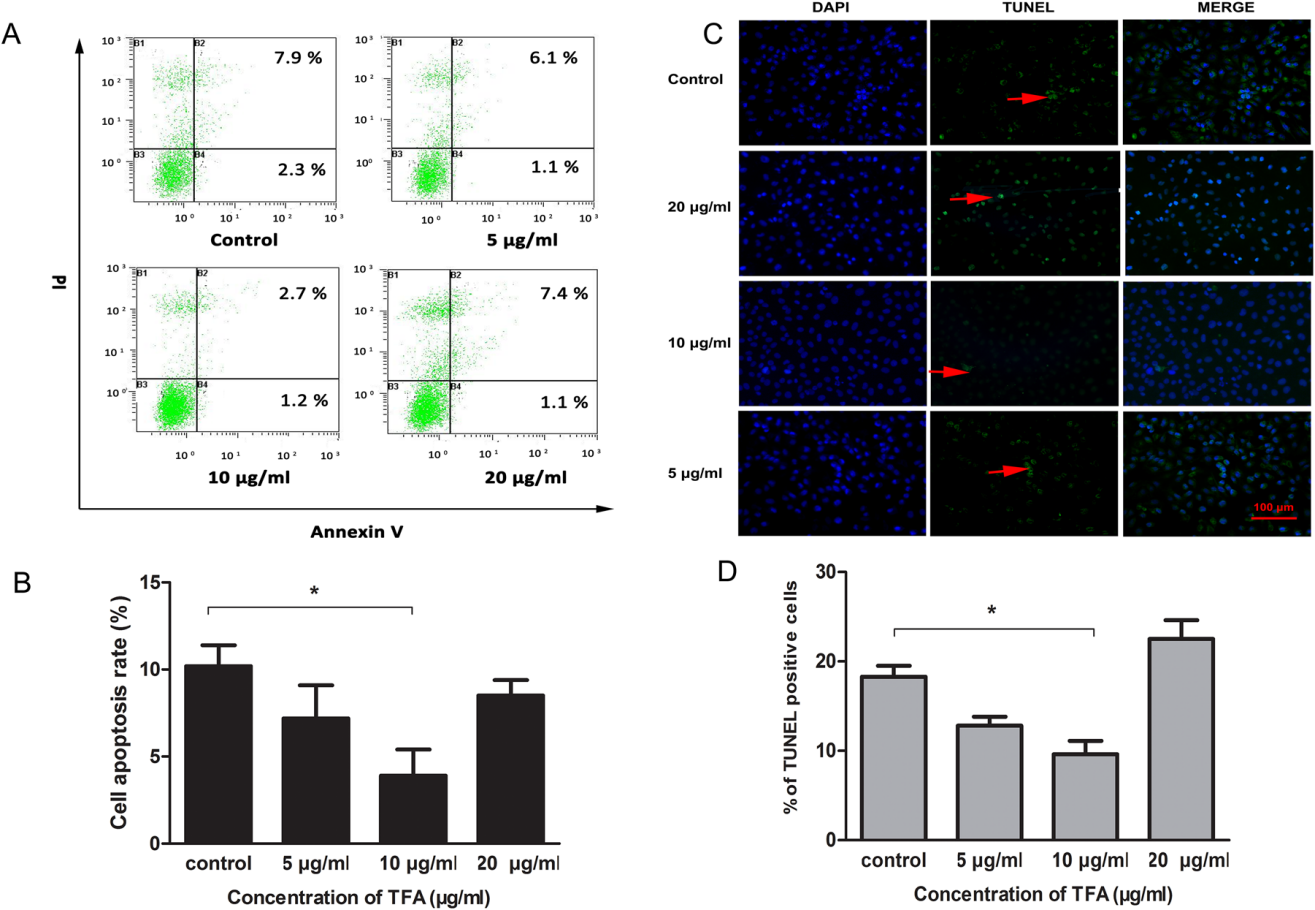
Effect of TFA on HUVECs apoptosis **(A)** The images of Annexin V / PI detection. HUVECs treated with TFA (5 μg/ml, 10 μg/ml, 20 μg/ml) were stained with Annexin V-FITC and PI and analyzed by FACS. **(B)** Percentage of apoptotic rate (including early and late apoptotic cells) determined by FACS. **(C)** Distribution of TUNEL-labeled apoptotic cells (arrow) in different groups (magnification 200×). TUNEL-positive cells were labeled with FITC (green) and nuclei were labeled with DAPI (blue). **(D)** Percentage of TUNEL positive cells. Data was expressed as means±SD. *p < 0.05 compared with control group.

TFA-treated HUVECs were characterized previously with the capacity of proliferation, migration and tube formation. We further evaluated the effect of TFA on HUVECs apoptosis. As shown in Figure 4A, the value in the lower right quadrant represented the apoptotic rate of Annexin V-positive and PI-negative, which characterized the early apoptotic stage. Moreover, the upper right quadrant containing double-stained cells represented the late phase of apoptosis. TFA intervention at 5 μg/ml, 10 μg/ml, 20 μg/ml resulted in a significant decrease in HUVECs apoptosis.

To gain insight into TFA effect on HUVECs apoptosis, TUNEL assay was performed. In the Figure 4C, TUNEL-positive cells were labeled with FITC (green) and nuclei were labeled with DAPI (blue). The number of TUNEL positive cells in the group treated with 10 μg/ml TFA was significantly less than in control group (*p<0.05, Figure 4D), which might explain to some extent the ability of TFA to attenuate HUVECs apoptosis.

### TFA enhanced VEGF and KDR expression in HUVECs

**Figure 5:**
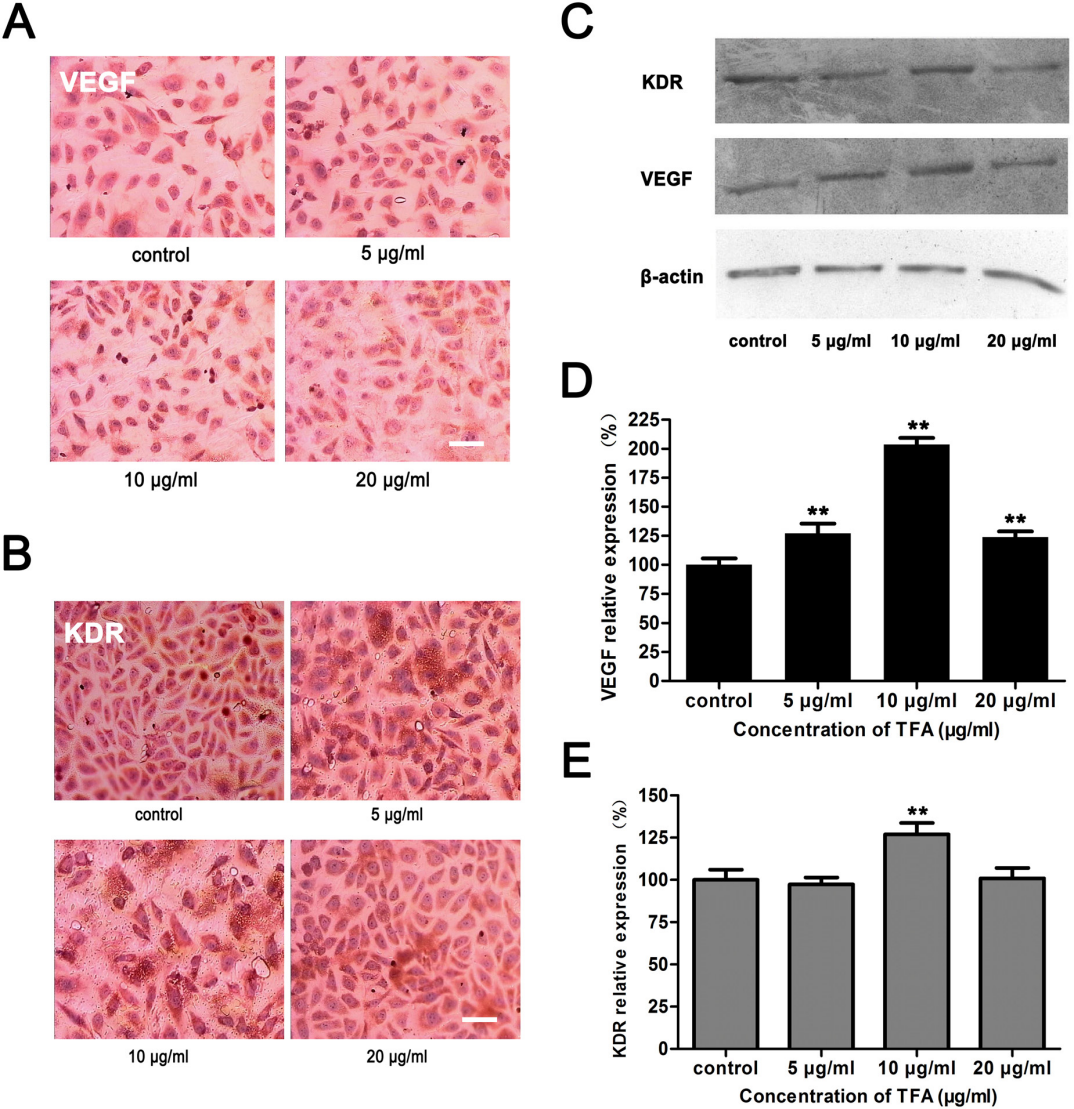
Effect of TFA on VEGF and KDR expression in HUVECs HUVECs were cultured with or without TFA to evaluate the influence on VEGF and KDR expression. **(A)** VEGF **(B)** KDR Immunocytochemistry images (magnification 400×). **(C)** Western blot and **(D) (E)** percentage of relative expression showing that TFA significantly increased VEGF expression and KDR expression only at TFA 10 μg/ml, which were consistent with the immunocytochemistry assay. Data are expressed as means±SD, **p<0.01 vs. control. Scale bars = 50 μm.

Vascular endothelial growth factor (VEGF) is a signaling protein produced by cells that stimulates vasculogenesis and angiogenesis. Kinase insert domain receptor (KDR), also known as vascular endothelial growth factor receptor 2 (VEGFR-2) appears to mediate almost all of the known cellular responses to VEGF [17]. As shown in Figure 5C and 5D, VEGF expression was significantly increased after TFA treatment at 5 μg/ml, 10 μg/ml and 20 μg/ml (**p < 0.01 vs. control group). Nevertheless, KDR expression displayed a statistically significant increase only at 10 μg/ml TFA (Figure 5E, **p <0.01 vs. control group). Immunocytochemistry images (magnification 400×) (Figure 5A and 5B) showed VEGF and KDR positive expression in TFA-treated HUVECs. These results suggested that TFA significantly increased VEGF expression and partially enhanced KDR expression.

## DISCUSSION

Total flavones of *Abelmoschus manihot* L. Medic (TFA) represent the major active component isolated from the traditional Chinese herb *Abelmoschus manihot* L. Medic [18]. Since previous studies had reported their successful use in the treatment of ischemic/reperfusion injuries and inflammatory diseases [7, 8], we therefore hypothesized that TFA could possess angiogenic properties. Angiogenesis is the physiological process through which new blood vessels form from pre-existing vessels [19]. In sprouting angiogenesis, endothelial cell proliferation, migration and tube formation are essential to the organized formation of vessel sprouts [20]. Hence, in the present study, we investigated these critical angiogenic processes. Firstly, we examined the effects of TFA on HUVECs viability and cell cycle to evaluate endothelial cell proliferation. The MTT and cell cycle assay indicated that TFA can prompt cells moving into DNA synthesis phase and keep HUVECs in a proliferative state. Secondly, we introduced transwell chamber culture system to quantify cell migration. It exhibited that the number of HUVECs penetrated to the lower membrane of the chamber increased after TFA treatment. Thirdly, we observed the effects of TFA on tube formation *in vitro* and *in vivo*. The results of a Matrigel Matrix assay demonstrated that TFA promoted HUVECs to form more complex and branched capillary-like structures. Furthermore, the results of CAM assay revealed that TFA-treated CAMs branched out into more multi-stage capillaries and more abundant neo-vasculatures. Together, our results in the present report demonstrated that TFA improved HUVECs proliferation, migration and tube formation, suggesting that TFA promoted the angiogenic processes.

In addition to cell proliferation, migration and tube formation, the Annexin V-FITC/PI double-staining flow cytometry and TUNEL assay were employed to explore the effect of TFA on HUVECs apoptosis. Annexin V-FITC/PI double-staining showed decreased apoptotic cells after TFA treatment. The result of TUNEL assay was consistent with flow cytometry analysis, further support that TFA treatment can reduce cell apoptosis.

In neovascularization, endothelial cell proliferation and migration represent the initial step, followed by the organization of cells into a network of tube-like structures and the formation of new capillaries [21, 22]. During these complex processes, vascular endothelial growth factor (VEGF) is considered the most significant proangiogenic factor since it induces cell proliferation and migration [23]. VEGF's normal function is to create new blood vessels during embryonic development, new blood vessels after injury, and new vessels (collateral circulation) to bypass blocked vessels. VEGFR-1, VEGFR-2 and VEGFR-3 are three VEGF receptors that have been recently identified. Among them, VEGFR-2 is the one involved in the process of angiogenesis [1]. Indeed, VEGFR-2, also known as kinase domain insert region (KDR), is considered the main receptor that mediates proliferation of endothelial cells following stimulation by VEGF-A. In this study, our results showed that TFA increased VEGF and KDR expression, although KDR was statistically enhanced only by one specific dose of TFA.

The partial upregulation of KDR could be explained by taxonomic features and mechanisms. The VEGF family in mammals is represented by five members: VEGF-A, VEGF-B, VEGF-C, VEGF-D and placenta growth factor (PGF). In addition, three VEGF receptors have been identified, such as VEGFR-1, VEGFR-2 and VEGFR-3. All members of the VEGF family stimulate cellular responses by binding to tyrosine kinase receptors (the VEGFRs) on the cell surface, causing them to dimerize and become activated through trans-phosphorylation. Each receptor binds several VEGF ligands but with different specificities. VEGFR-1, also known as flt-1, is the binding site of VEGF-A and VEGF-B. It is a high-affinity VEGFR that occurs as a splice variant giving rise to a secreted soluble extra-cellular domain, fms-like tyrosine kinase-1 (sflt1, also known as soluble VEGF receptor-1) which is a potent antagonist of VEGF. It is also thought to modulate VEGFR-2 signaling [24]. Furthermore, VEGFR-1 acts as a dummy/decoy receptor, sequestering VEGF from VEGFR-2 binding. VEGFR-2 (KDR/Flk-1) appears to mediate almost all of the known cellular responses to VEGF [17]. It is thought to be the main receptor that mediates proliferation of endothelial cells following stimulation by VEGF-A [1]. VEGFR-3 (Flt4) induces lymphangiogenesis in response to VEGF-C and VEGF-D, which mediates perpetual action and function of ligands on target cells.

As previously noted, VEGFR-2 (KDR) mediates the effect of VEGF on endothelial cell migration, proliferation, differentiation, and survival in addition to vessel permeability and dilation. However, other VEGFRs may be involved in the process of angiogenesis. Indeed, recent reports described the diverse roles of VEGFR-1. Adult transgenic mice lacking the PGF gene showed a defective response to ischemia, wound healing, inflammation and cancer, despite the normal development of embryonic vasculature. These effects could not be corrected by other ligands binding to flt1 such as VEGF-B, suggesting a specific interaction between PGF and VEGFR-1. Moreover, PGF was also able to induce arteriogenesis in adult rabbits and more effectively than a ligand specific for VEGFR-2 [25, 26]. PGF is a member of the VEGF sub-family. It is a key molecule in angiogenesis and vasculogenesis. Furthermore, PGF expression within human atherosclerotic lesions is associated with plaque inflammation and neovascular growth [27, 28]. Taken together, these evidences, together with our results showing KDR enhancement by one specific dose of TFA, suggest that, since KDR is the main receptor through which VEGF mediates its biological effects in endothelial cells, other VEGFRs, such as VEGFR-1, may participate in the process of angiogenesis. On the basis of this result, more experimental assay such as protein microarray should be performed to further explore the process of angiogenesis induced by TFA. Therefore, our future study will focus on the specific TFA ingredient(s) in promoting angiogenic effect to evaluate its specific mechanism of action, with the aim to develop a novel treatment choice for wound healing and ischemic/reperfusion injuries.

## MATERIALS AND METHODS

### Preparation of TFA

*Abelmoschus Manihot* L. Medic were collected from Jiangyan of Jiangsu province, China. TFA was extracted from the flowers of *Abelmoschus Manihot* by the Department of Chinese Materia Medica, Nanjing University of Chinese Medicine, Nanjing, China. A total of 500g of *Abelmoschus Manihot* flowers was immersed in 8000 ml 75% ethanol for 1 h. The mixture was refluxed for 1 h at 90 °C and filtered by analytical filter paper. The extracts were evaporated by rotary evaporation under vacuum at 60 °C [29]. The “drug extract” ratio (DER) of *Abelmoschus manihot* ethanol extract is within 4.0 – 4.5: 1 [30]. The main active components of *Abelmoschus manihot* L.Medic were hyperoside, isoquercitrin, hibifolin, quercetin-3’-O-glycoside, quercetin, myricetin and rutin. HPLC fingerprint of Abelmoschus manihot L. Medic extract can be referred to the previously published paper [31].

### Cells and cell culture

Human umbilical vein endothelial cells (HUVECs) were purchased from American Type Culture Collection (ATCC, Manassas, VA, USA). The complete growth medium used for this cell line was ATCC-formulated F-12K Medium (Catalog No. 30-2004, ATCC, Manassas, VA, USA) supplemented with 0.1 mg/ml heparin (Sigma-Aldrich, St. Louis, Mo, USA), 0.03 mg/ml endothelial cell growth supplement (ECGS; Sigma-Aldrich, St. Louis, Mo, USA) and 10% (v/v) heat-inactivated fetal bovine serum (FBS; GIBCO, USA). Cell cultures were carried out in a 5% CO_2_ humidified atmosphere incubator at 37°C (Heracell 150i, Heraeus, Langenese, Germany).

### Testing the TFA working concentration

The working concentration of TFA was tested based on cell viability. Cell viability was determined by 3-[4, 5-Dimethylthiazol-2-yl]-2, 5-diphenyltetrazoliumbromide assay (MTT; Sigma-Aldrich, St. Louis, Mo, USA). HUVECs were seeded in sterile 96-well plates at a density of 1×10^4^ cells/ml. After 24 h, the adherent cells were treated with TFA at concentrations ranging from 0.1 μg/ml to 160 μg/ml and incubated for 48 h and 72 h. Subsequently MTT solution (20 μl/well) was added and the cells were incubated at 37°C, 5% CO_2_ for 4 h. Then, the culture medium was removed and 150 μl of DMSO (Sigma-Aldrich, St. Louis, Mo, USA) were added to each well. After 10 min of shaking at room temperature (RT), the absorbance was measured at 490 nm by a microplate reader (BioTek, Winooski, VT, USA).

Based on the results on Figure 1A, 5 μg/ml, 10 μg/ml, and 20 μg/ml TFA represented the optimal working concentrations were used in our subsequent experiments.

### Cell cycle analysis

HUVECs were seeded in sterile six-well plates at a density of 3×10^5^ cells/ml and incubated with TFA at different concentrations (0 μg/ml, 5 μg/ml, 10 μg/ml and 20 μg/ml) for 72 h. Subsequently, the harvested cells were fixed with 70% (v/v) cold ethanol at 4°C overnight. Cells were washed with cold phosphate buffered saline (PBS) twice and resuspended in 500 μl staining buffer containing 25 μl propidium iodide (PI) solution and 10 μl RNase A (C1052, Beyotime, Shanghai, China) at 37°C in the dark for 15 min. After that, the cells were analyzed by flow cytometry (FACS, BD Biosciences, San Jose, CA, USA).

### Cell migration assay

Cell migration was quantified using the Boyden chamber assay [32]. HUVECs migration was performed in a Transwell system (Millipore, Billerica, MA, USA) composed of 8 μm pore size polycarbonate filter inserts in each well of the 24-well plates. Prior to the assay, the lower side of the membranes of the transwell was coated with fibronectin as an adhesive substrate. Cells were deprived of fetal bovine serum overnight. Subsequently, serum-starved cells were trypsin-harvested and washed twice in PBS. Based on the Boyden chamber principle, HUVECs (1×10^5^) suspended in 100 μl of serum-free medium were inoculated in each upper well of the transwell chambers, while 600 μl of culture medium containing 10% FBS and TFA at different concentrations (0 μg/ml, 5 μg/ml, 10 μg/ml, 20 μg/ml) were added to each lower chamber. Each concentration was performed in duplicate. After 12 h incubation, the non-migrated cells on the upper side of the inserts were removed using a cotton swab. The lower side of the membranes were fixed with 95% ethanol and stained with 0.1% crystal violet. The number of migrated cells attached to the lower side of the inserts was counted in 5 random fields using an optical microscope (Olympus, Tokyo, Japan) at 100× magnification.

### Tube formation assay

Aliquots of 300 μl of Matrigel (Catalog No.356234, Corning, Bedford, MA, USA) were added to each well of 24-well plates, followed by incubation at 37 °C for 30 min. HUVECs (1×10^5^) were suspended in 0.5% FBS culture medium containing TFA at different concentrations (0 μg/ml, 5 μg/ml, 10 μg/ml, 20 μg/ml). Subsequently, HUVECs were seeded at a density of 1 × 10^5^ cells per well in 24-well Matrigel-coated plates (Corning Costar, NY, USA) and incubated for 6 h. Tube branch and length were measured in three random microscopic fields at 100× magnification (Olympus, Tokyo, Japan) and quantified using ImageJ analysis software. The assays were performed three times with duplicate independent samples.

### Vessel formation in chick chorioallantoic membrane (CAM)

The effect of TFA on angiogenesis *in vivo* was evaluated using a chick chorioallantoic membrane (CAM) model [33, 34]. Ten fertilized chicken eggs (45∼55 g) in each group were incubated at 37°C and 70% humidity for 7 days. On the eighth day of incubation, a tiny square window was opened in each shell. Sterile gelatin sponges saturated with TFA at different concentrations (5 μg/ml, 10 μg/ml, 20 μg/ml) and normal saline (NS, control group) were placed on the areas of preexisting vessels for extra 3 d. After 3 d of incubation, all the CAMs of each group were fixed with 5 drops of 1:1 v/v mixture of methanol and acetone for 15 min. The number of blood vessel enclosed in the rings of 1 and 5 mm diameters around the gelatin sponges was photographed and counted under the microscope (Olympus, Tokyo, Japan). The experiments were approved by the experimental ethics committee of Nanjing University of Chinese Medicine.

### Cell apoptosis assay

HUVECs apoptosis was quantified by an Annexin V / PI detection kit (Vazyme, Nanjing, Jiangsu, China) according to the manufacturer’s instructions and analyzed by flow cytometry (BD Biosciences, San Jose, CA, USA). Cells were seeded in sterile 6-well plates at a density of 3×10^5^ cells/ml and incubated with TFA at different concentrations (0 μg/ml, 5 μg/ml, 10 μg/ml, 20 μg/ml). After incubation of 72 h, the harvested cells were suspended in binding buffer at a density of 1×10^6^ cells / ml. Then, 5 μl of Annexin V- Fluorescein isothiocyanate (FITC) and 5 μl of PI were added to 100 μl of cell resuspension solution and the mixture was placed in the dark at RT for 15 min. 400 μl of binding buffer was added and the cells were analyzed by FACS. At least 10,000 cells per sample were analyzed.

Terminal deoxynucleotidyl transferase-mediated dUTP nick end-labeling assay (TUNEL; Vazyme, Nanjing, Jiangsu, China) was also performed to observe the effect of TFA on HUVECs apoptosis. HUVECs were seeded on sterile coverslips in 6-well culture plates at a density of 3×10^5^ cells/ml to allow attachment. When cells reached 60% confluence, the medium was removed. Then, HUVECs were treated with TFA at different concentrations (0 μg/ml, 5 μg/ml, 10 μg/ml, 20 μg/ml). Afterwards, the cells were fixed with 4% paraformaldehyde for 25 min at 4°C. Fixative was removed and coverslips were washed twice with PBS. Then sufficient volume of the permeabilization reagent was added to completely cover the coverslips. After washed twice with PBS, the coverslips were incubated with 1× Equilibration Buffer for 15 min at room temperature. 100 μL of terminal deoxynucleotidyl transferase (TdT) reaction cocktail was added to each coverslip and the solution was allowed to spread completely over the surface in a humidified chamber for 60 min at 37°C. Each coverslip was washed twice with PBS and then incubated with 4',6-diamidino-2-phenylindole (DAPI) for 5 min at room temperature. The percentage of TUNEL positive cells was calculated under a fluorescent microscope (Olympus, Tokyo, Japan) at 200× magnification.

### Western blot analysis

The expressions of vascular endothelial growth factor (VEGF) and Kinase Insert Domain Receptor (KDR, also known as VEGFR-2) were analyzed by Western blot. HUVECs were plated into sterile 6-well plates at a density of 3×10^5^ cells/ml to allow attachment. When cells reached 60% confluence, TFA at different concentrations (0 μg/ml, 5 μg/ml, 10 μg/ml, 20 μg/ml) were added to the plates and incubated for 72h. Each concentration group was performed in triplicate. Cells were lysed on ice and centrifuged at 10,000×g for 10 min at 4°C. The supernatants were collected and the concentrations of total protein were detected using a BCA kit (Beyotime, Shanghai, China). Equivalent amounts of protein (30 μg protein per lane) were separated using 10% sodium dodecyl sulfate- polyacrylamide gel electrophoresis (SDS-PAGE) for 2 h at 100 V and transferred to a polyvinylidene fluoride membrane (PVDF; Millipore, Billerica, MA, USA). Afterwards the membrane was blocked using 5% (w/v) nonfat milk. The blots were incubated overnight at 4°C with primary antibodies against VEGF (1:200, Santa Cruz, Dallas, TX, USA), KDR (1:200, Santa Cruz, Dallas, TX, USA), and beta-actin (Santa Cruz, Dallas, TX, USA). After incubation with secondary horseradish peroxidase–conjugated antibody (1:1000, Beyotime, Shanghai, China) for 2 h at RT, the proteins in the PVDF membranes were visualized by enhanced chemiluminescence (ECL, Pierce, Rockford, IL, USA). The intensity of the protein bands was quantitatively analyzed using ImageJ software and normalized with the intensity of beta-actin.

### Immunocytochemistry

VEGF and KDR were detected by immunocytochemistry in HUVECs treated with TFA (0 ug/ml, 5 μg/ml, 10 μg/ml, 20 μg/ml). Briefly, HUVECs were seeded on sterile coverslips in 6-well culture plates at a density of 3×10^5^ cells/ml to allow attachment. When cells reached 60% confluence, the medium was removed. Then, HUVECs were treated with TFA at different concentrations (0 μg/ml, 5 μg/ml, 10 μg/ml, 20 μg/ml) and incubated for 72h. Each group was performed in triplicate. Afterwards, the cells were fixed with 10% formalin for 10 min. Each coverslip was treated with 3% (v/v) H_2_O_2_ for 15 min to block the activity of endogenous peroxidase and blocked with 5% (w/v) bovine serum albumin for 10 min. The medium was discarded and each coverslip was stained with VEGF antibody (1:200, Santa Cruz, Dallas, TX, USA), or KDR antibody (1:200, Santa Cruz, Dallas, TX, USA) at 4°C overnight prior to incubation with HRP-conjugated secondary antibody for 30 min. The antibody binding sites were visualized by incubation with 3-Amino-9-ethylcarbazole (AEC) solution and counterstained with hematoxylin. One hundred cells were counted in three randomly selected fields for each coverslip with 400× magnification. A score from 0 to 3 was conferred to identify the staining intensity (3 = strongest expression).

### Statistical analysis

Data were analyzed using SPSS 16.0 statistical package. Multiple comparisons were performed by one-way analysis of variance (ANOVA) followed by Student *t*-test. All results were expressed as mean±SD. Values of *p* < 0.05 were considered statistically significant.

## CONCLUSION

Our results demonstrated that TFA, as the major active component isolated from the traditional Chinese herb *Abelmoschus manihot* L. Medic, could activate HUVECs proliferation and migration, and attenuate cell apoptosis. Moreover, TFA could enhance angiogenic ability of HUVECs *in vitro* and CAM *in vivo* by up-regulating the expression of VEGF and KDR.
